# Greenspace and Depression in LASI

**DOI:** 10.1093/geroni/igaf122.044

**Published:** 2025-12-31

**Authors:** Samer Atshan

**Affiliations:** Center for Economic and Social Research, Los Angeles, California, United States

## Abstract

Greenspaces, such as parks, gardens, and natural vegetation, are increasingly recognized as protective against mental health challenges among older adults. However, findings have primarily emerged from high-income, urbanized contexts, and evidence from low- and middle-income countries remains limited. Using data from the Longitudinal Aging Study in India-Diagnostic Assessment of Dementia (LASI-DAD), this study examines the relationship between exposure to greenspace and depressive symptoms as assessed by the CESD-10 scale. Our analysis specifically explores how this relationship varies across urban and rural contexts. Preliminary results indicate that in rural areas, respondents residing in greener environments reported lower scores on positive affect items of the CESD-10 scale, suggesting fewer experiences of positive emotions such as happiness and optimism. We explore potential explanations including socioeconomic influences in rural settings related to agricultural labor, experiences of social isolation and loneliness, and limited access to healthcare services. This study highlights the importance of contextualizing environmental determinants of mental health in aging research and demonstrates the value of harmonized environmental data in generating nuanced insights for culturally relevant mental health interventions.

